# 5-Azacitidine Partially Resets the Subcellular Localization of YAP in Human Bone Marrow-Derived Mesenchymal Stem Cells

**DOI:** 10.3390/cells15060524

**Published:** 2026-03-16

**Authors:** Hidehito Takayama, Hisashi Kishi, Gen Kobashi

**Affiliations:** Department of Public Health, Dokkyo Medical University School of Medicine, Mibu 321-0293, Japan

**Keywords:** mechanotransduction, mesenchymal stem cell, 5-azacitidine, epigenetics, mechanical memory, extracellular matrix, YAP

## Abstract

**Highlights:**

**What are the main findings?**

- Transient 5-azacitidine partially resets mechanically sustained nuclear YAP localization in human bone marrow-derived MSCs.
- RNA-seq indicates broad downregulation of extracellular matrix-related genes after 5-azacitidine exposure.

**What are the implications of the main findings?**

- Epigenetic modulation may represent a simple strategy to attenuate culture-induced mechanical memory during ex vivo expansion.
- This approach may improve consistency of mechanosensitive YAP regulation during MSC preparation for therapeutic applications.

**Abstract:**

Mesenchymal stem cells (MSCs) sense biophysical cues from their microenvironment, which regulate cytoskeletal organization and the nuclear–cytoplasmic distribution of the mechanotransducer Yes-associated protein (YAP), thereby shaping cellular behavior. Prolonged ex vivo culture on non-physiologically rigid substrates induces persistent nuclear YAP localization, a phenomenon often referred to as mechanical memory. We therefore examined whether transient epigenetic modulation could modulate YAP subcellular localization in human bone marrow-derived MSCs. Treatment with the DNA methyltransferase inhibitor 5-azacitidine (5-Aza) shifted YAP localization toward the cytoplasm in MSCs, without overt changes in pluripotency marker expression or neural differentiation capacity. RNA sequencing revealed broad down-regulation of extracellular matrix (ECM)-related genes following 5-Aza treatment. Independent suppression of ECM production via TGF-β signaling similarly promoted cytoplasmic YAP localization. When subsequently transferred to soft substrates, 5-Aza–treated MSCs restored YAP relocalization despite prior expansion on stiff surfaces. Together, these findings suggest that transient 5-Aza treatment can partially alleviate mechanically induced YAP regulation associated with mechanical memory. Thus, simple and transient administration of 5-Aza may offer a practical means to improve the quality of MSCs during ex vivo expansion for cell-based therapies.

## 1. Introduction

Cells are influenced by diverse external stimuli, including chemical factors, mechanical stress, light, and temperature [1]. These signals collectively regulate homeostasis, proliferation, motility, differentiation, and senescence. Among these stimuli, mechanical forces are sensed through mechanosensitive modules, enabling cells to respond to physical cues in their microenvironment [2,3,4]. During development, cells rely on physical cues to guide morphogenesis, lineage specification, and tissue patterning, ultimately orchestrating organ size and shape in developing embryos [5,6].

Mechanical cues are transduced into intracellular signals through adhesion complexes and cytoskeletal tension. At the cell–extracellular matrix (ECM) interface, integrin α/β heterodimers cluster into focal adhesions [7], which transmit mechanical signals to the Hippo pathway, where MST1/2 and LATS1/2 kinases regulate Yes-associated protein (YAP) phosphorylation and subcellular localization.

Cells sense ECM stiffness by actively applying traction forces and probing the resulting matrix deformations, linking displacement magnitude to substrate stiffness [2,8]. In addition to bulk stiffness, surface topography, geometric constraints and integrin ligand spacing modulate mechanotransduction [9,10]. These physical parameters influence cytoskeletal organization and downstream signaling pathways, thereby regulating cellular behavior. Understanding how cells sense and respond to substrate stiffness is important not only for developmental and homeostatic processes but also for biomedical applications, such as implant integration and tissue engineering, where inappropriate material stiffness can lead to undesired cellular behaviors [11].

Yes-associated protein is a transcriptional coactivator that binds to TEAD family transcription factors and serves as a central mechanotransducer [12]. YAP/TAZ shuttle between the cytoplasm and the nucleus, with cytoplasmic localization being inactive and nuclear localization enabling transcriptional activation [13,14]. Dysregulated or persistent nuclear YAP has been implicated in pathological processes, including tumorigenesis and tissue fibrosis [7], highlighting the importance of tight regulation of its subcellular localization.

In mesenchymal stem cells (MSCs), YAP predominantly localizes to the nucleus when cultured on stiff substrates, whereas soft substrates promote cytoplasmic retention [15]. This stiffness-dependent localization recapitulates the physiological diversity of tissue mechanics in vivo, ranging from the soft brain to rigid bone. Engler et al. demonstrated that, in the absence of chemical signal (i.e., cytokines/growth factors), substrate stiffness directs MSC lineage specification, with upregulation of osteogenic markers on differentiation on stiff matrices (25–40 kPa), myogenic markers on intermediate matrices (8–17 kPa), and neurogenic markers on soft matrices (0.1–1 kPa) [16]. During embryonic development, YAP activity is generally high, supporting tissue growth and morphogenesis, whereas in adult cells its activity is maintained at lower levels [17,18]. YAP activity can transiently increase in response to tissue injury and is frequently overactivated in cancers [19], highlighting its context-dependent regulation.

Several studies have reported that stem cells can “remember” past physical inputs from their extracellular environment, a phenomenon termed mechanical memory [20,21,22,23]. Such persistence suggests that transient mechanical cues can be converted into long-lasting intracellular states. These mechanically imprinted states are thought to contribute to cellular heterogeneity during long-term culture and may underlie the variable therapeutic outcomes observed in stem cell-based interventions.

MSCs are among the most widely studied stem cell populations for regenerative medicine, with numerous clinical trials exploring their applications in tissue repair and immunomodulation [24,25,26,27]. Standard tissue culture plates (TCPs) have an elastic modulus near 10^9^ Pa, far exceeding physiological stiffness, which can induce persistent nuclear YAP localization. For example, MSCs expanded on stiff substrates may show enhanced osteogenic differentiation, and prolonged culture can reduce stemness and alter differentiation capacity. These changes complicate large-scale in vitro expansion [23,24,28,29].

In this study, we aimed to explore a way to regulate YAP localization toward a more physiologically relevant state by using human bone marrow-derived MSCs (hBM-MSCs). Prolonged culture on stiff substrates is associated with sustained nuclear YAP localization, suggesting that transient mechanical inputs may be converted into stabilized transcriptional states. Because epigenetic modifications are known to stably regulate gene expression, we hypothesized that epigenetic mechanisms may contribute to the persistence of mechanically imprinted YAP activity. Accordingly, we examined whether transient modulation of DNA methylation using 5-azacitidine, one of the well-characterized DNA methyltransferase inhibitors, could influence mechanically sustained YAP localization in MSCs.

## 2. Materials and Methods

### 2.1. Cell Culture

Human bone marrow-derived mesenchymal stem cells were purchased from Lonza (Basel, Switzerland). hBM-MSCs were cultured on the 10 cm tissue culture plate (100 × 17 mm EasYDish TC Surface, Catalogue number: 150464, Thermo Fisher Scientific, Waltham, MA, USA) and 6- or 24-well cell culture dish (Catalogue number: 92406, 92424, TPP, Trasadingen, Switzerland) in Dulbecco’s modified Eagle’s medium (DMEM, high glucose; Catalogue number: 11965092, Thermo Fisher Scientific), supplemented with 10% fetal bovine serum (FBS, Cytiva, Piscataway, NJ, USA), 1 ng/mL basic fibroblast growth factor (bFGF; 130-093-840, Miltenyi Biotec, Gladbach, Germany), and 100 μg/mL kanamycin (Catalogue number: 15160054, Thermo Fisher Scientific) at 37 °C in a humidified atmosphere containing 5% CO_2_, and the medium was changed every two days.

For epigenetic modulation, cells were treated with 5-azacitidine (5-Aza; Nacalai Tesque, Kyoto, Japan). Unless otherwise stated, cells in the treatment group were exposed to 5 μM 5-Aza for 120 h. The concentration (5 μM) was selected based on prior in vitro studies employing low-micromolar concentrations of 5-Aza in vitro [30,31], while the exposure duration (120 h) was determined based on preliminary time-course observations and previous reports describing treatments with DNMT inhibitors [32].

### 2.2. Immunostaining

hBM-MSCs cultured on the tissue culture plate or soft matrix plate (see Section 2.8) were washed three times with 1× PBS and fixed with 4% paraformaldehyde (PFA) for 10 min at room temperature. After fixation, cells were washed three times with 1× PBS and then blocked with blocking buffer (0.8% (*w*/*v*) BlockAce (KAC, Kyoto, Japan), 5% (*w*/*v*) BSA, 0.3% (*v*/*v*) Triton X-100, 1× PBS) for 30 min [33]. Cells were then incubated overnight at 4 °C with a rabbit anti-YAP antibody (1:200; Catalogue number: 14074s, Cell Signaling Technology, Danvers, MA, USA) diluted in buffer A (2 g/L BlockAce, 10 g/L BSA, 0.3% (*v*/*v*) Triton X-100 in 1× PBS). F-actin was stained with phalloidin conjugated to iFluor488 (1:1000; Catalogue number: 20549, Cayman Chemical, Ann Arbor, MI, USA) in buffer A for 30 min in the dark. After washing, cells were incubated for 1 h with a fluorescently labeled secondary antibody (1:500; goat anti-rabbit IgG antibody DyLight549, Catalogue number: GTX213110-05, GeneTex, Irvine, CA, USA) and DAPI (Catalogue number: D9542, Sigma-Aldrich, St. Louis, MO, USA) diluted in buffer B (0.4% (*v*/*v*) Triton X-100 in 1× PBS).

Fluorescence images were acquired using a BZ-X710 fluorescence microscope (Keyence, Osaka, Japan) equipped with PlanApo λ 10× (NA 0.45) or 20× (NA 0.75) objectives. Images were captured in monochrome mode using LED illumination and standard DAPI, GFP, and TRITC filter sets. Images were acquired at 16-bit depth without pixel binning. Exposure times were optimized to avoid pixel saturation and were maintained constant within each experimental dataset. Raw images were used for quantitative analysis without LUT modification.

### 2.3. Quantification of YAP Nucleo-Cytoplasmic Localization

Quantification of YAP localization was performed using ImageJ (version 1.54g; NIH). For each condition, individual cell boundaries were manually delineated based on phalloidin staining to define cell shape, and nuclear regions were defined using DAPI staining. Regions of interest (ROIs) corresponding to the whole cell and nucleus were saved for further YAP quantification. Background fluorescence was estimated by averaging measurements from three cell-free regions per image and subtracted from all measurements. Total YAP fluorescence intensity (red channel) was quantified within whole-cell and nuclear ROIs after background correction by multiplying the mean fluorescence intensity by the corresponding ROI area. Cytoplasmic YAP intensity was calculated by subtracting the nuclear signal from the whole-cell signal. The nucleo-cytoplasmic YAP ratio was calculated as the ratio of total nuclear to cytoplasmic YAP fluorescence intensity. Cells with overlapping boundaries or ambiguous segmentation were excluded from the analysis. Fluorescence images were acquired using a 10× or 20× objective depending on the experimental setup. Quantitative comparisons were performed only within datasets acquired under identical imaging conditions.

### 2.4. Neural Differentiation

Neural differentiation was induced according to previously reported protocol [34], with minor modifications, by culture in Neurobasal medium containing 1% FCS, 1× B27 supplement, and cAMP-elevating compounds (1-methyl-3-isobutylxanthine, forskolin, and 8-CPT-cAMP) together with dexamethasone and valproic acid. In the present study, morphological changes were observed within 24–48 h, and cells were collected at 48 h after induction for subsequent analyses.

### 2.5. Quantitative PCR (qPCR)

Cells were harvested by trypsinization and washed with 1× PBS twice. Cell pellets obtained by centrifugation at 400× *g* for 5 min were applied to NucleoSpin RNA XS (Macherey-Nagel, Dueren, Germany) to extract total RNA. A cDNA library was prepared using ReverTra Ace qPCR RT master mix (Toyobo, Osaka, Japan) under the following conditions: 37.5 °C for 15 min, 50 °C for 5 min, and 98 °C for 5 min. qPCR quantification was performed using Thunderbird Probe qPCR Mix (Toyobo). The condition of the thermal cycler (QuantStudio 3, Thermo Fisher, Waltham, MA, USA) was as follows: initial denaturation at 94 °C for 20 s, 40 cycles at 95 °C for 3 s to denature, and 60 °C for 30 s for extension. qPCR quantification was performed by Thunderbird Probe qPCR Mix (Toyobo, Osaka, Japan) with *GAPDH* as a housekeeping gene. Primers (Thermo Fisher, Catalogue number: 4331182) used in this study are shown in Table 1.

### 2.6. RNA-Seq Analysis

After adhesive cell culturing for 120 h, cells were harvested by trypsin and RNA-seq library preparation was carried out using TruSeq mRNA standard (Illumina, San Diego, CA, USA). The expression profile analysis was performed by NovaSeq 6000 sequencer using a 100 bp single-end read.

The resulting FASTQ files were analyzed with the web-based platform OlvTools (https://olvtools.com/rnaseq/introduction) (accessed on 8 March 2026) for differential expression analysis (DEG) and Gene ontology analysis. Differential expression analysis in OlvTools was performed using an edgeR-based workflow. Gene expression counts were normalized using the trimmed mean of M-values (TMM) method, and *p*-values were adjusted for multiple testing using the Benjamini–Hochberg false discovery rate (FDR). Genes with FDR < 0.05 were considered differentially expressed. To assess potential batch effects, principal component analysis (PCA) was examined to evaluate overall sample clustering. A volcano plot was generated using Python (version 3.14.0).

### 2.7. The Regulation of YAP Localization Mediated by the TGF-β Signaling Pathway

For adhesive culture in 24-well TCP, hBM-MSCs were seeded in the culture medium with TGF-β1 (Catalogue number: 100-B-001, R&D systems, Minneapolis, MN, USA) at 5 ng/mL final concentration or 10 μM SB431542 (Catalogue number: 1614/1, R&D systems), a TGF-β signaling pathway inhibitor, for 96 h.

### 2.8. Cell Culture on the Soft Matrix Plate

To observe the cell behavior on a soft matrix with a stiffness of 0.5 kPa, a polydimethylsiloxane (PDMS) coated tissue culture dish (CytoSoft, Advanced BioMatrix, Carlsbad, CA, USA) was utilized [35]. Because of a lack of cell adhesion property of PDMS gel, the bovine type I collagen (CosmoBio, Tokyo, Japan) was covalently attached to surface-modified PDMS via the amine group. Collagen solution (100 μg/mL, 1× DPBS) was adjusted to pH 7.4 with 1 N NaOH and applied to CytoSoft wells for 30 min. The wells were washed twice with 1× PBS, after which the cell suspension was added.

### 2.9. ELISA

Adherent cells were harvested using a cell scraper, and the protein levels of total YAP and phosphorylated YAP (Ser127) were quantified using the PathScan Total YAP sandwich ELISA kit and PathScan Phospho-YAP (Ser127) sandwich ELISA kit (Cell Signaling Technology), according to the manufacturer’s instructions. Collagen type I alpha 2 (COL1A2) encodes a major component of type I collagen, one of the most abundant structural proteins in the ECM [36,37]. The protein level of COL1A2 in conditioned medium (including PBS washes) was measured using an Enzyme-linked Immunosorbent Assay Kit for Collagen Type I Alpha 2 (USCN Life Science, Wuhan, China) using a plate reader.

### 2.10. Statistics

Data are presented as mean ± standard deviation (SD). Statistical significance was assessed using the Mann–Whitney U test. A *p*-value less than 0.05 was considered statistically significant. Significance levels are indicated in the figures as follows: * *p* < 0.05, and *** *p* < 0.001.

## 3. Results

### 3.1. 5-Azacitidine Shifts YAP Localization of hBM-MSC Toward the Cytoplasm

In order to discover a method to regulate the YAP localization in mesenchymal stem cells, we focused on an approach based on the addition of small-molecule reagents. Reports indicate that YAP can exhibit prolonged nuclear retention following sustained mechanical stimulation, suggesting that once established, its subcellular distribution can persist beyond the timescale of acute mechanical signaling [7,20,23]. Such persistence is also evident in pathological conditions: in fibrotic tissues, YAP remains aberrantly enriched in the nucleus, and its transcriptional activity is sustained over extended periods, consistent with the chronic course of scar formation [15]. These observations imply that mechanically induced states may remain stable over multi-day or even multi-passage culture. Because epigenetic modifications are known to stably maintain transcriptional programs in mammalian cells [38], we reasoned that epigenetic mechanisms might contribute to the long-term retention of mechanically imprinted states. Accordingly, we tested whether pharmacological epigenetic regulators could reverse persistent nuclear YAP and restore a more physiological localization pattern.

**Figure 1:**
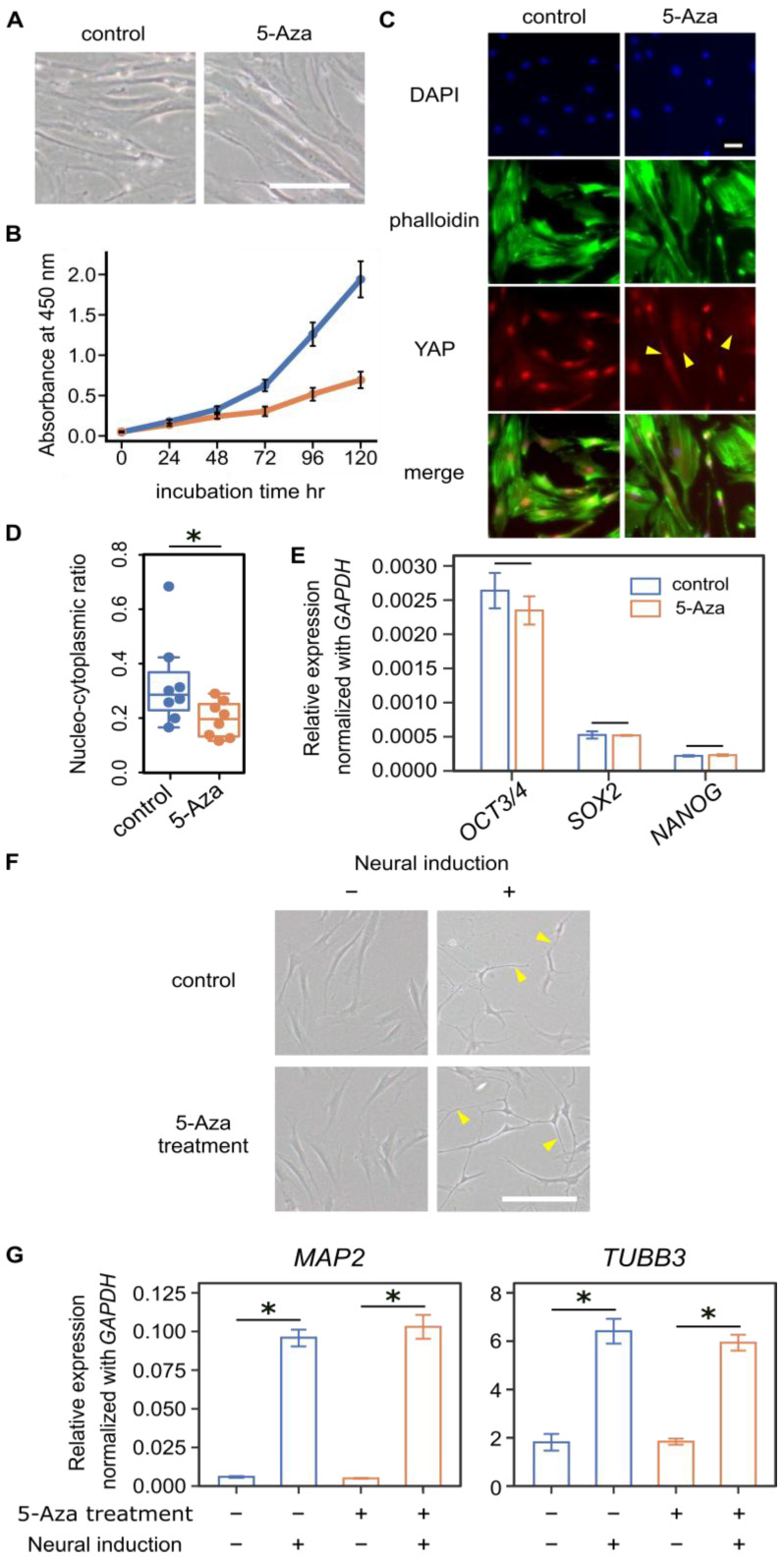
5-Azacitidine (5-Aza) shifts YAP localization toward the cytoplasm in hBM-MSCs. (**A**) Morphology of hBM-MSCs treated with 5 μM 5-Aza for 120 h and control cells. Scale bar = 100 μm. (**B**) Cell growth under 5-Aza treatment up to 120 h, measured by CCK-8. Blue line indicates control cells, and orange line indicated 5-Aza-treated cells. Data are presented as means ± SEM (*n* = 3). (**C**) Representative immunofluorescence images of hBM-MSCs: DAPI (blue) for nuclei, phalloidin (green) for F-actin, and YAP (red). Arrows indicate cells exhibiting diffuse cytoplasmic YAP localization. Scale bar = 50 μm. (**D**) Effect of 5-Aza on YAP nucleo-cytoplasmic ratio in hBM-MSCs (*n* = 8 per group). Each dot represents an individual cells. 5-Aza treatment significantly reduced nuclear YAP localization. (**E**) Relative mRNA expression of pluripotency markers *OCT3/4*, *SOX2*, and *NANOG*, normalized to *GAPDH*. No significant differences were observed between control and 5-Aza-treated cells (*n* = 3). (**F**) Neural differentiation was induced in control and 5-Aza-treated cells for 48 h. Arrows indicate representative elongated cellular processes following neural induction. Scale bar = 100 μm. (**G**) Expression of neural markers (*MAP2* and *TUBB3*) was quantified in control and 5-Aza-treated cells, with or without neural induction (*n* = 4). A significant difference was observed after 48 h of neural induction. In (**D**,**E**,**G**), statistical significance was assessed using the Mann–Whitney U test (* denotes *p* < 0.05).

hBM-MSCs were cultured on tissue culture plates with or without 5-azacitidine (5-Aza), a DNA methyltransferase inhibitor, for 120 h. The overall morphology of 5-Aza-treated cells was similar to that of control cells (Figure 1A). Quantitative analysis of circularity and aspect ratio revealed no marked differences between groups (Supplementary Figure S1), although cell proliferation was reduced (Figure 1B). In control cells, YAP staining was predominantly nuclear, with weak cytoplasmic signals detectable in many cells (Figure 1C). In contrast, 5-Aza-treated cells exhibited heterogeneous YAP localization: while nuclear-dominant YAP staining was still observed in a subset of cells, many cells displayed a diffuse and predominantly cytoplasmic distribution of YAP (Figure 1C, arrows). Image analysis showed that the nuclear-to-cytoplasmic ratio was significantly reduced by 5-Aza treatment (Figure 1D). Because stem cells have the capacity to differentiate, we examined the expression of pluripotent markers *OCT3/4* (also known as *POU5F1*), SRY-box transcription factor 2 (*SOX2*), and *NANOG* by qPCR. None of these markers showed significant differences between control and 5-Aza-treated cells (Figure 1E). To assess differentiation potential, hBM-MSCs were induced toward a neural lineage for 48 h. After induction, both 5-Aza-treated and control cells exhibited elongated processes (Figure 1F, arrows). Neural induction significantly increased *MAP2* and *TUBB3* expression within each group (Figure 1G).

### 3.2. RNA-Seq Analysis Revealed Reduced ECM-Related Gene Expression Following 5-Aza Treatment

**Figure 2:**
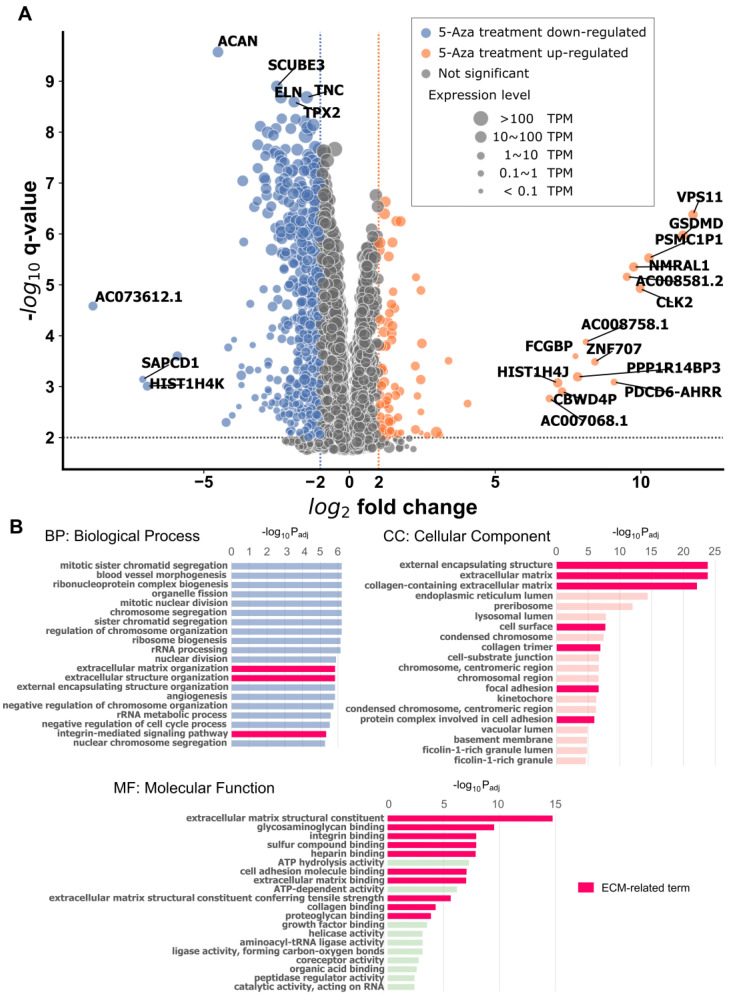
Gene expression profiles of 5-Aza-treated hBM-MSC analyzed by RNA-seq. (**A**) Volcano plot showing differential gene expression following 5-Aza treatment. Genes with fold change > 4 and q-value < 0.01 are indicated in color (orange, up-regulated; blue, down-regulated). (**B**) Gene ontology (GO) enrichment analysis highlighting extracellular matrix (ECM)-related terms in Biological Process (BP), Cellular Component (CC), and Molecular Function (MF) categories. Bars are colored according to GO categories: blue for BP, light pink for CC, and green for MF.

Since 5-Aza shifted YAP localization epigenetically by inhibiting DNA methylation in hBM-MSCs, we next investigated its impact on global gene expression profiles by RNA-seq after 120 h of treatment compared with untreated controls (Figure 2A).

Gene ontology (GO) enrichment analysis revealed that ECM-related GO terms were significantly affected by 5-Aza treatment (Figure 2B). For the Biological Process, enriched terms included extracellular matrix organization (GO:0030198) and extracellular structure organization (GO:0043062). For the Cellular Component, enriched terms included external encapsulating structure (GO:0030312), extracellular matrix (GO:0031012), collagen-containing extracellular matrix (GO:0062023), cell surface (GO:0009986), and collagen trimer (GO:0005581). For the Molecular Function, enriched terms included extracellular matrix structural constituent (GO:0005201) and extracellular matrix structural constituent conferring tensile strength (GO:0030020).

In addition, ECM-interacting pathways were also enriched. For example, the integrin-mediated signaling pathway (GO:0007229) was detected in the Biological Process, whereas cell–substrate junction (GO:0030055), focal adhesion (GO:0005925), and protein complex involved in cell adhesion (GO:0098636) were enriched in the Cellular Component category. Similarly, glycosaminoglycan binding (GO:0005539), integrin binding (GO:0005178), sulfur compound binding (GO:1901681), heparin binding (GO:0008201), cell adhesion molecule binding (GO:0050839), extracellular matrix binding (GO:0050840), collagen binding (GO:0005518), and proteoglycan binding (GO:0043394) were enriched in the Molecular Function, suggesting altered cell-ECM interactions.

At the gene level, many ECM-related transcripts—including aggrecan, elastin, fibronectin, fibrillin, and some collagen chains—were significantly down-regulated by 5-Aza treatment (Table 2). Among these, *COL1A1* and *COL1A2* exhibited the largest reductions (Supplementary Figure S2).

### 3.3. Effect of TGF-β Signaling-Mediated ECM Modulation on YAP Localization

Surprisingly, 5-Aza treatment clearly altered ECM synthesis, as shown in Section 3.2. We therefore investigated whether changes in the ECM are tightly linked to YAP localization. Because ECM integrity and stiffness are key determinants of YAP nuclear localization, alterations in ECM synthesis or organization—such as those induced by 5-Aza—are expected to influence YAP activity and subcellular distribution.

ECM synthesis itself is regulated by a complex interplay of biochemical and mechanical cues. Mechanical stimuli, including tension, compression, and shear stress, are sensed by mechanoreceptors such as integrins, which trigger intracellular signaling cascades that ultimately influence ECM turnover, reflecting a dynamic balance emerging from concomitant ECM synthesis and degradation, and determining the net ECM abundance.

In parallel, a broad range of cytokines and growth factors modulate ECM composition in a context-dependent manner, reflecting the highly dynamic nature of ECM regulation. Cytokines and growth factors, including TGF-β [39,40,41,42,43,44,45], platelet-derived growth factor (PDGF) [44,46,47], fibroblast growth factor (FGF) [48,49], and insulin-like growth factor (IGF) [43,50,51], fine-tune ECM synthesis and remodeling. Among these biochemical regulators, TGF-β acts as a central node linking extracellular signals to transcriptional programs governing ECM production. TGF-β, in particular, is critically involved in fibrosis and drives excessive deposition of ECM components such as collagen, fibronectin, and proteoglycans.

**Figure 3:**
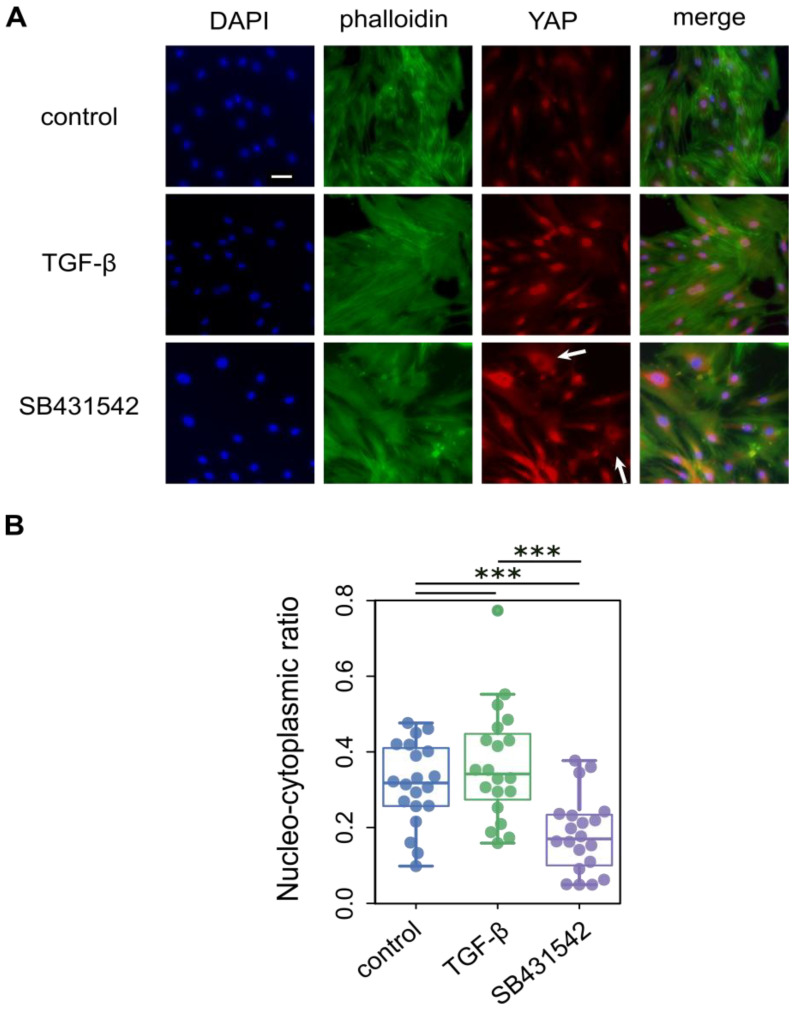
Effect of TGF-β signaling on YAP localization in hBM-MSCs. (**A**) hBM-MSCs were treated with 5 ng/mL TGF-β and 10 μM SB431542, the potent TGF-β signaling inhibitor. Immunofluorescence staining was performed using DAPI (blue), phalloidin (green) for F-actin, and YAP (red). Scale bar = 50 μm. Arrows indicate cells showing cytoplasmic exclusion of YAP. (**B**) Effect of TGF-β signaling on the YAP nucleo-cytoplasmic ratio in hBM-MSCs (*n* = 20 per group). Each dot represents an individual cell, and colored dots indicate experimental conditions (blue, control; green, TGF-β-treated cells; purple, SB431542-treated cells). TGF-β treatment did not significantly affect YAP localization, whereas inhibition of TGF-β signaling by SB431542 induced a marked shift in YAP from the nucleus to the cytoplasm. Significant differences between groups are indicated by *** (*p* < 0.001, Mann–Whitney U test).

To experimentally examine whether ECM reduction affects YAP localization, we manipulated TGF-β signaling in hBM-MSCs. Cells were treated with TGF-β1 (5 ng/mL) to promote ECM production or with SB431542 (10 μM), a potent TGF-β receptor antagonist, to suppress downstream signaling. hBM-MSCs were cultured on a tissue culture plate for 96 h. For each condition, cells in 24-well plate were analyzed by immunofluorescence, assessing YAP nucleo-cytoplasmic distribution (Figure 3A). An effect of TGF-β1 treatment at 5 ng/mL was limited and slightly increased nucleo-cytoplasmic ratio of YAP (*p* = 0.337, Figure 3B), likely reflecting the inherent mechanobiological influence of the stiff tissue culture plate. On the contrary, a potent antagonist of the TGF-β receptor, SB431542, which inhibits downstream signaling with high efficiency, substantially shifted YAP localization toward the cytoplasm compared with untreated control cells (*p* = 0.000758). Notably, even a subset of cells showed marked exclusion of YAP from the nucleus, indicating a robust effect of ECM suppression on YAP translocation. These observations highlight that ECM abundance and composition, mediated via TGF-β signaling, are critical determinants of YAP subcellular localization, linking biochemical and mechanical cues to transcriptional regulation in MSCs.

Together, these results indicate a mechanistic model in which epigenetic modulation by 5-Aza indirectly influences YAP localization by altering ECM synthesis. This effect is supported by complementary experiments using TGF-β-dependent signaling, which independently links ECM abundance to YAP localization by integrating extracellular cues with intracellular transcriptional pathways. This framework suggests that manipulation of ECM, either via epigenetic drugs or targeted modulation of TGF-β signaling, may provide a strategy to attenuate cellular mechanical memory and optimize stem cell properties for therapeutic applications. Furthermore, our findings provide a conceptual bridge connecting long-term epigenetic modifications to acute mechanotransduction events, illustrating how the extracellular environment and intracellular signals converge to regulate stem cell behavior.

### 3.4. Recovery of YAP Localization of MSC on the Soft Matrix by 5-Aza Treatment

For cell therapy applications, MSCs require repeated in vitro expansion, during which prolonged exposure to stiff culture substrates can stabilize mechanically imprinted states. Strategies to reset such mechanical memory have included the use of micro-patterned substrates composed of alternating soft and stiff regions created by photolithography [52]. Although effective, these approaches require specialized fabrication and handling. We therefore examined whether a simpler chemical strategy based on transient epigenetic modulation could facilitate recovery of YAP localization in response to substrate compliance.

**Figure 4:**
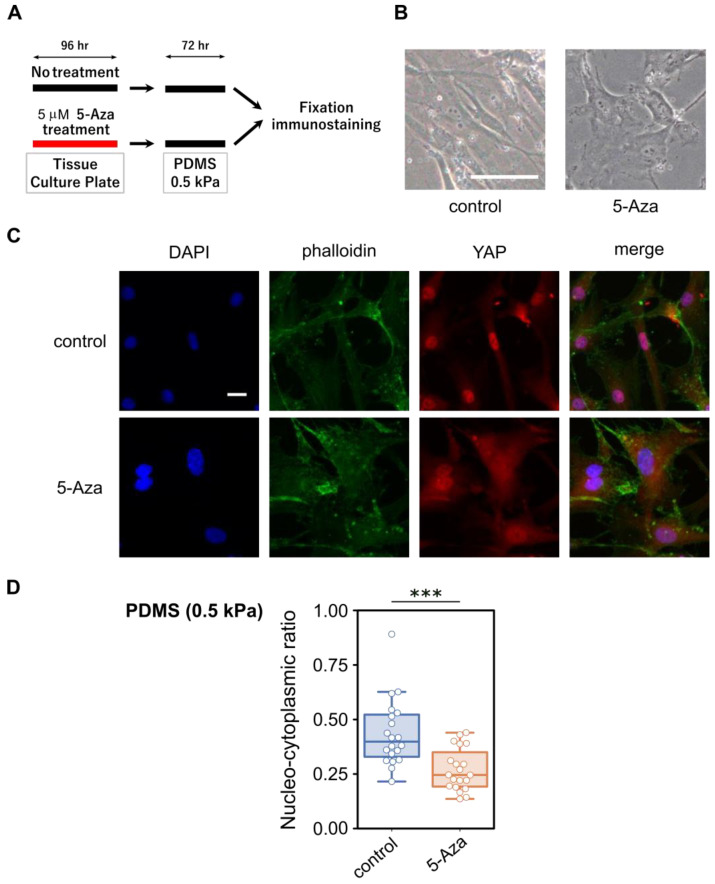
Effect of transient 5-Aza treatment on YAP localization in hBM-MSCs cultured on soft substrates. (**A**) Schematic representation of the experimental design. hBM-MSCs were treated with 5 µM 5-Aza on tissue culture plates (TCPs) and subsequently reseeded onto soft PDMS substrates (elastic modulus: 0.5 kPa) for analysis. (**B**) Representative phase-contrast images showing cell morphology after 72 h of culture on soft PDMS. Scale bar = 100 μm. (**C**) Representative immunofluorescence images showing DAPI (blue), phalloidin (green), and YAP (red), acquired using a 20× objective. Scale bar = 20 μm. (**D**) Quantification of the nucleo-cytoplasmic YAP ratio (*n* = 20 per group). Each dot represents an individual cell. Colored dots indicate experimental conditions (blue, control; orange, 5-Aza-treated cells). Significant differences between groups are indicated by *** (*p* < 0.001, Mann–Whitney U test).

Specifically, we treated hBM-MSCs cultured on conventional TCP with 5 μM 5-Aza for 96 h. After treatment, cells were thoroughly washed three times with 1× PBS to remove residual 5-Aza and subsequently transferred to soft PDMS-gel-coated culture dishes with an elastic modulus of 0.5 kPa, providing a more physiologically relevant mechanical environment, in the absence of 5-Aza (Figure 4A). This experimental design enabled us to isolate the effect of transient epigenetic modulation from continuous chemical exposure, allowing us to test whether transient epigenetic modification could overcome previously established mechanical memory without the need for complex patterned substrates. During subsequent culture on the soft PDMS substrate, 5-Aza pre-treated cells showed only minor morphological differences compared with controls, appearing slightly less elongated and somewhat more rounded, with no overt changes in overall morphology or phenotypic abnormalities (Figure 4B). Untreated cells largely retained nuclear YAP localization even on the soft matrix plate, consistent with the notion that long-term culture on stiff substrates induces a persistent nuclear retention of YAP (Figure 4C). Indeed, the nuclear localization pattern of YAP in control cells on soft PDMS was largely indistinguishable from that of hBM-MSCs maintained continuously on TCP. In contrast, a substantial proportion of 5-Aza pre-treated cells displayed cytoplasmic YAP localization, indicating a partial reversal of mechanically imprinted nuclear retention.

Quantitative analysis of nucleo-cytoplasmic YAP ratios further confirmed these observations (Figure 4D). Images for Figure 1C and Figure 3A were acquired using a 10× objective, whereas images for Figure 4C were acquired using a 20× objective. Because images were obtained under different optical and substrate conditions (TCP versus PDMS), absolute nucleo-cytoplasmic ratios may vary across experimental settings. Therefore, comparisons were made within datasets acquired under identical imaging conditions. Moreover, because this experimental setup involved cell detachment, re-plating, and re-adhesion onto compliant PDMS substrates, variability in YAP intensity was observed, likely reflecting heterogeneity in cell spreading and cytoskeletal organization on soft substrates. Nevertheless, 5-Aza-treated cells consistently exhibited a significant shift toward lower nucleo-cytoplasmic YAP ratios relative to untreated controls, supporting the conclusion that transient epigenetic modulation facilitates YAP relocalization in response to substrate compliance.

### 3.5. Time-Course Analysis of YAP-Related Events

To quantify the temporal relationship between ECM dynamics and YAP regulation, we performed a time-course analysis of type I collagen abundance and YAP phosphorylation. Type I collagen was used as a representative ECM component, while YAP phosphorylation was assessed as a biochemical indicator closely linked to its nuclear–cytoplasmic shuttling. Phosphorylation of YAP promotes its cytoplasmic retention through interaction with 14-3-3 proteins. hBM-MSCs were treated with 5-Aza, and COL1A protein levels and the ratio of phosphorylated YAP to total YAP were measured by ELISA at multiple time points from 0 to 120 h.

**Figure 5:**
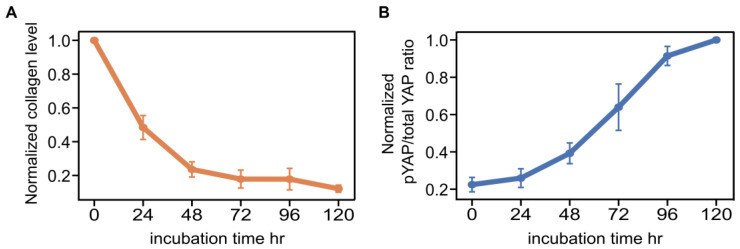
Time-course analysis of ECM levels and YAP phosphorylation ratio following transient 5-Aza treatment. hBM-MSCs were treated with 5-Aza and analyzed over a 0–120 h time course. (**A**) Normalized collagen level relative to 0 h decreased over time. (**B**) Normalized pYAP/total YAP ratio relative to the 120 h value increased over time. Data are presented as mean ± SEM (*n* = 3).

Time-course analysis revealed that collagen levels decreased rapidly within the first 24 h (Figure 5A), whereas the pYAP/total YAP ratio increased more gradually over the same period (Figure 5B). This temporal pattern indicates that ECM depletion occurs rapidly and precedes major changes in YAP phosphorylation, supporting the hypothesis that ECM reduction is an upstream cue promoting cytoplasmic YAP translocation. Preliminary experiments confirmed that collagen levels and YAP phosphorylation remained stable over time in untreated control cells.

The gradual increase in YAP phosphorylation also suggests the presence of regulatory feedback loops that may modulate YAP localization over time. While ECM reduction precedes cytoplasmic YAP translocation along the time course, other intracellular or extracellular factors may act synergistically or independently to fine-tune YAP activity. These findings indicate the complexity of mechanotransduction networks.

**Figure 6:**
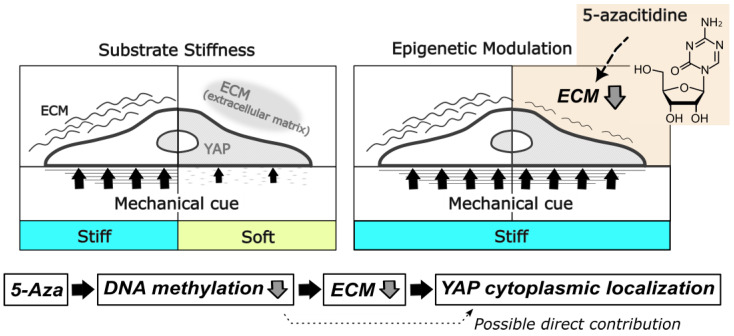
Conceptual model summarizing the findings of this study. Reduced ECM production is associated with cytoplasmic YAP localization. A potential direct contribution of DNA hypomethylation to cytoplasmic YAP localization is indicated by a dashed arrow.

In summary, our time-course analyses suggest a model in which transient exposure to 5-Aza rapidly reduces ECM abundance, which in turn drives cytoplasmic translocation of YAP. This mechanism likely contributes, at least in part, to partial resetting of mechanically induced YAP regulation during previous rounds of MSC expansion on stiff substrates (Figure 6). Nevertheless, the potential involvement of alternative pathways, such as transcriptional regulation by nuclear YAP and other modulators yet to be identified, remains to be clarified.

## 4. Discussion

In this study, we demonstrated that transient 5-Aza exposure shifts YAP localization toward the cytoplasm in human bone marrow-derived MSCs. This shift was accompanied by reduced expression of extracellular matrix-related genes and partial resetting of persistent nuclear YAP localization induced by stiff substrates. Time-course analysis revealed that ECM reduction precedes changes in YAP phosphorylation. These findings suggest that transient epigenetic modulation can modify mechanically imprinted YAP regulation.

Although ECM reduction appears to contribute to YAP localization, the precise mechanism by which 5-Aza suppresses ECM gene expression has not been fully elucidated. Because 5-Aza alters DNA methylation, it may directly influence the expression of mechanosensitive genes. Future epigenomic analyses, such as whole-genome bisulfite sequencing (WGBS) [53], may help identify methylation changes associated with ECM downregulation and altered YAP localization.

MSCs are heterogeneous and contain multiple subpopulations, including multilineage-differentiating stress-enduring (Muse) cells [54]. Such heterogeneity may influence how individual MSCs respond to epigenetic modulation and mechanotransductive cues. Cell-to-cell variability in YAP localization observed in this study may therefore reflect intrinsic variability in cell cycle state, epigenetic landscape, or local ECM organization within the MSC population.

This study was conducted under conventional 2D culture conditions, which do not fully recapitulate the mechanical complexity of native 3D tissues. Future studies using 3D culture systems, including decellularized ECM models [55,56] and bioreactors employing microcarriers or cell aggregates for large-scale cell expansion, will be necessary to evaluate the physiological relevance of these findings within more complex mechanical microenvironments. In addition, altered tissue stiffness in pathological conditions such as fibrosis [57] may further influence YAP regulation.

Although no overt cytotoxicity by 5-Aza was observed in this study, prolonged or repeated exposure to epigenetic modifiers may influence DNA damage responses, cellular senescence, or apoptosis. Thus, systematic evaluation of 5-Aza concentration, exposure duration, frequency and dosing interval will be necessary in future studies to define conditions that achieve epigenetic modulation without compromising cellular integrity. DNMT inhibitors have been reported to enhance interferon signaling and antigen presentation pathways in cancer cells, thereby increasing immune recognition [58]. Although such immune-related effects were not evaluated in MSCs in the present study, careful assessment of immune-related gene expression and functional immunogenicity will be required. In addition, mitochondrial dynamics and metabolic state have been linked to stemness regulation in several stem cells [59]. DNMT inhibitors, including 5-Aza, may also exert RNA-dependent effects that influence global protein synthesis and potentially mitochondrial function [60]. Therefore, careful optimization of dose and exposure duration will be important to minimize unintended metabolic or bioenergetic alterations before considering clinical translation.

Beyond these safety and translational considerations, several limitations should be acknowledged. The durability of YAP relocalization beyond the treatment period remains to be determined. In addition, 5-Aza exerts broad epigenomic effects that may influence pathways unrelated to mechanotransduction. Moreover, whether YAP relocalization is directly mediated by DNA methylation changes at specific regulatory loci also remains unclear. While ECM reduction appears to contribute to YAP cytoplasmic translocation, additional regulatory layers may exist. Transient epigenetic modulation may also influence protein–protein interactions involving YAP, which contains intrinsically disordered regions (IDRs) [61]. Further studies will be required to clarify the specific contribution of DNA methylation to YAP regulation.

In conclusion, transient 5-azacitidine exposure modulates YAP subcellular localization in human bone marrow-derived MSCs and is associated with reduced ECM-related gene expression. These findings indicate that epigenetic intervention can alter persistent YAP localization induced by prolonged mechanical cues. Although the precise molecular mechanisms remain to be clarified, the present study provides experimental evidence linking DNA methylation-dependent modulation to YAP-associated mechanotransduction in MSCs. Future studies integrating epigenomic and functional analyses will help define how epigenetic interventions improve mechanosensitive cellular states.

## 5. Conclusions

In this study, we demonstrate that 5-azacitidine (5-Aza) promotes a shift of the mechanotransducer Yes-associated protein (YAP) from the nucleus to the cytoplasm without inducing major changes in stemness. Our results indicate that epigenetic modulation by 5-Aza is associated with reduced extracellular matrix abundance, followed by redistribution of YAP from the nucleus to the cytoplasm. These findings suggest that transient 5-Aza treatment can partially alleviate mechanically imprinted regulation of YAP acquired during prolonged culture on stiff substrates. Collectively, our study highlights that simple pharmacological epigenetic modulation may represent a useful strategy to improve the functional quality of mesenchymal stem cell cultures expanded for therapeutic applications.

## Figures and Tables

**Table 1:** Primers used for qPCR.

Target Gene	Primer Number
*GAPDH*	Hs02786624_g1
*OCT3/4*	Hs04260367_gH
*NANOG*	Hs04399610_g1
*SOX2*	Hs01053049_s1

**Table 2:** A list of down-regulated genes following 5-Aza treatment.

Gene Name	Protein Name	log_2_FC *	FDR †	ECM
*ACAN*	Aggrecan core protein	−4.51315	3.63 × 10^−6^	Yes
*SCUBE3*	Signal peptide, CUB domain and EGF-like domain containing 3	−2.4944	6.90 × 10^−6^	
*TNC*	Tenascin C	−1.46753	6.90 × 10^−6^	Yes
*ELN*	Elastin	−2.35206	6.90 × 10^−6^	Yes
*TPX2*	TPX2 microtubule nucleation factor	−1.90001	6.90 × 10^−6^	
*COL11A1*	Collagen type XI alpha 1 chain	−2.32307	8.70 × 10^−6^	Yes
*IGFBP5*	Insulin-like growth factor binding protein 5	−1.84931	8.70 × 10^−6^	
*ABI3BP*	ABI family member 3 binding protein	−1.53783	8.70 × 10^−6^	associated
*COL8A1*	Collagen type VIII alpha 1 chain	−1.24074	8.70 × 10^−6^	Yes
*TROAP*	Trophinin associated protein	−3.06017	8.70 × 10^−6^	
*ARL6IP1*	ARL6 interacting reticulophagy regulator 1	−1.45339	8.70 × 10^−6^	
*CENPF*	Centromere protein F	−2.52177	8.70 × 10^−6^	
*HJURP*	Holliday junction recognition protein	−2.01351	8.70 × 10^−6^	
*COL12A1*	Collagen type XII alpha 1 chain	−1.35354	8.70 × 10^−6^	Yes
*CCNB1*	Cyclin B1	−2.8046	9.15 × 10^−6^	
*AURKA*	Aurora kinase A	−2.61032	9.54 × 10^−6^	
*COL1A1*	Collagen type I alpha 1 chain	−1.50081	9.54 × 10^−6^	Yes
*PLK1*	Polo like kinase 1	−3.14732	1.25 × 10^−5^	
*CALM3*	Calmodulin 3	−1.17438	1.25 × 10^−5^	
*CDC20*	Cell division cycle 20	−2.80053	1.25 × 10^−5^	
*COL5A1*	Collagen type V alpha 1 chain	−1.04355	1.25 × 10^−5^	Yes
*FN1*	Fibronectin 1	−0.50376	1.25 × 10^−5^	Yes
*CENPE*	Centromere protein E	−2.19964	1.25 × 10^−5^	
*CEMIP*	Cell migration inducing hyaluronidase 1	−1.78874	1.25 × 10^−5^	associated
*BIRC5*	Baculoviral IAP repeat containing 5	−2.53458	1.25 × 10^−5^	
*SPARC*	Secreted protein acidic and cysteine-rich	−0.87914	1.25 × 10^−5^	associated
*FBN1*	Fibrillin 1	−0.87827	1.26 × 10^−5^	Yes

* Fold Change compared to control. † False Discovery Rate (Benjamini–Hochberg adjusted *p*-value).

## Data Availability

All data generated or analyzed during this study are available from the corresponding author upon reasonable request.

## Supplementary Materials

The following supporting information can be downloaded at: https://www.mdpi.com/article/10.3390/cells15060524/s1. Figure S1: Quantitative analysis of cell morphology in control and 5-azacitidine-treated hBM-MSCs. Figure S2: RNA-seq expression levels of collagen genes in control and 5-azacitidine-treated hBM-MSCs.
