# Supplementary material for: 5-Azacitidine Partially Resets the Subcellular Localization of YAP in Human Bone Marrow-Derived Mesenchymal Stem Cells
Source: Cells. 2026 Mar 16;15(6):524. doi: 10.3390/cells15060524 (PMC13025356; doi:10.3390/cells15060524)
Supplement: Supplementary file 1 [file cells-15-00524-s001.zip › cells-4130628-supplementary-revised/Supplementary_Materials_revised.pdf]

Supplementary Figure S1. Quantitative analysis of cell morphology in control and 5-azacitidine-treated hBM-MSCs.

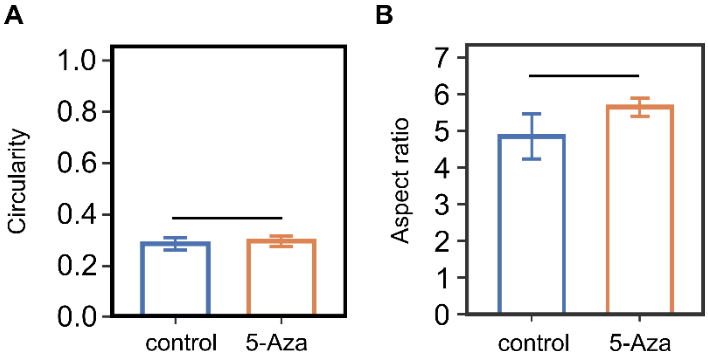

For each biological replicate (n = 3 per condition), 50 individual cells were analyzed. Circularity and aspect ratio were quantified using ImageJ. Data are presented as mean ± SEM from three independent biological replicates.

Supplementary Figure S2. RNA-seq expression levels of collagen genes in control and 5-azacitidine-treated hBM-MSCs.

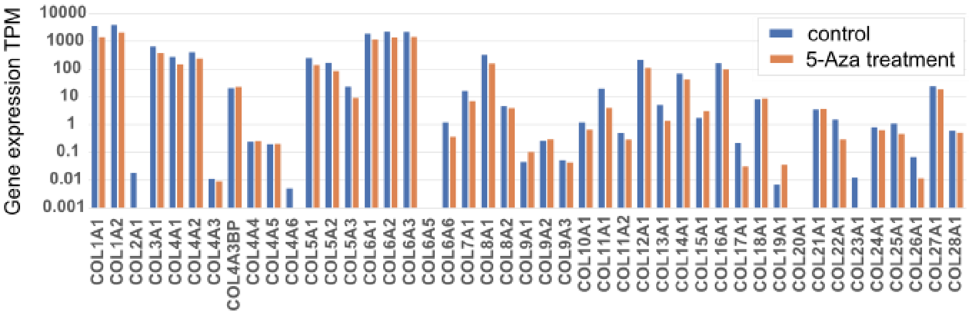

Gene expression levels in control and 5-azacitidine-treated cells are shown as transcripts per million (TPM).
