# Supplementary figures and images for: 5-Azacitidine Partially Resets the Subcellular Localization of YAP in Human Bone Marrow-Derived Mesenchymal Stem Cells
Source: Cells. 2026 Mar 16;15(6):524. doi: 10.3390/cells15060524 (PMC13025356; doi:10.3390/cells15060524)

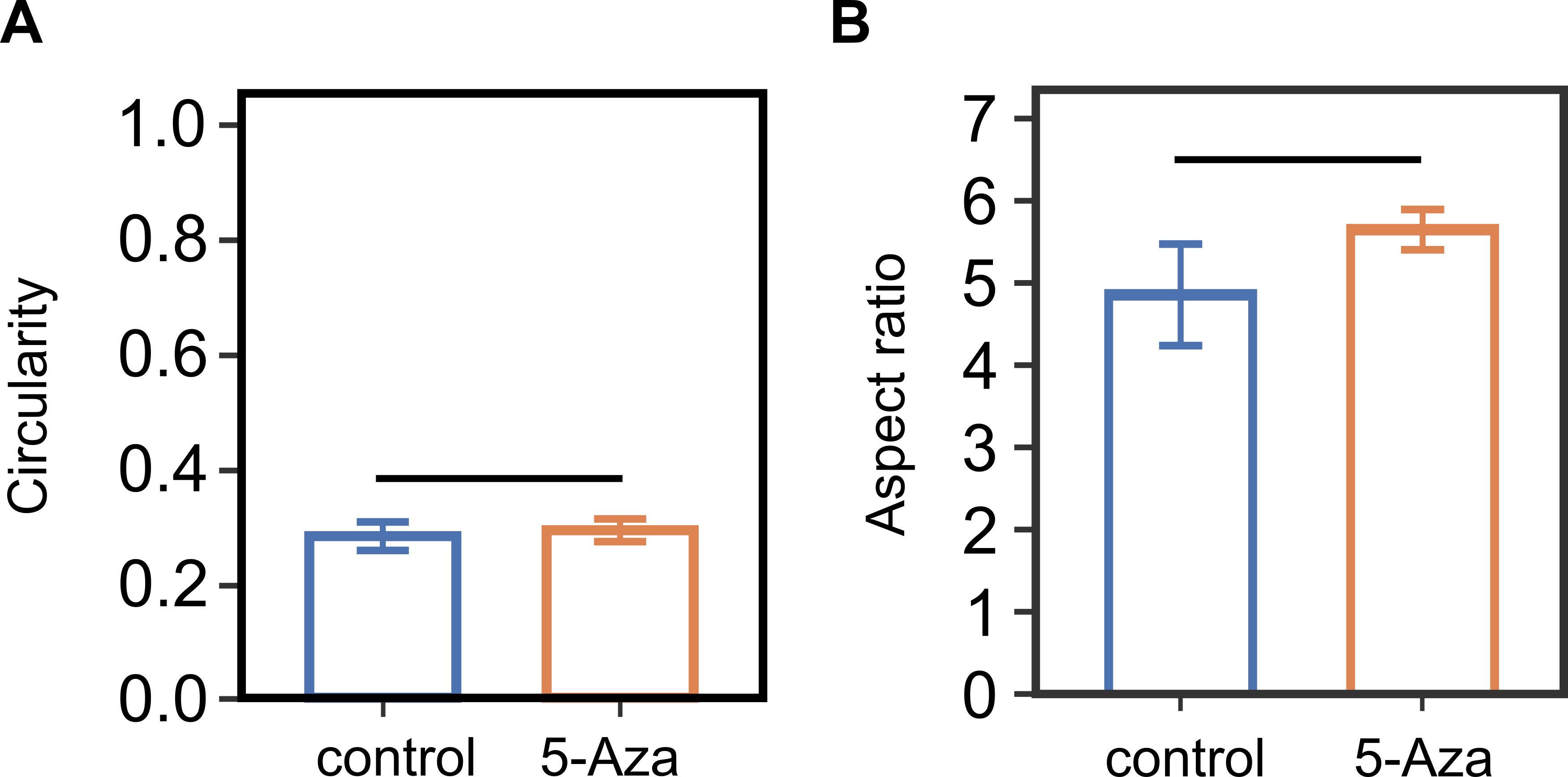

Supplement: Supplementary file 1 [file cells-15-00524-s001.zip › cells-4130628-supplementary-revised/Supplementary_Figure_S1_revised.png]

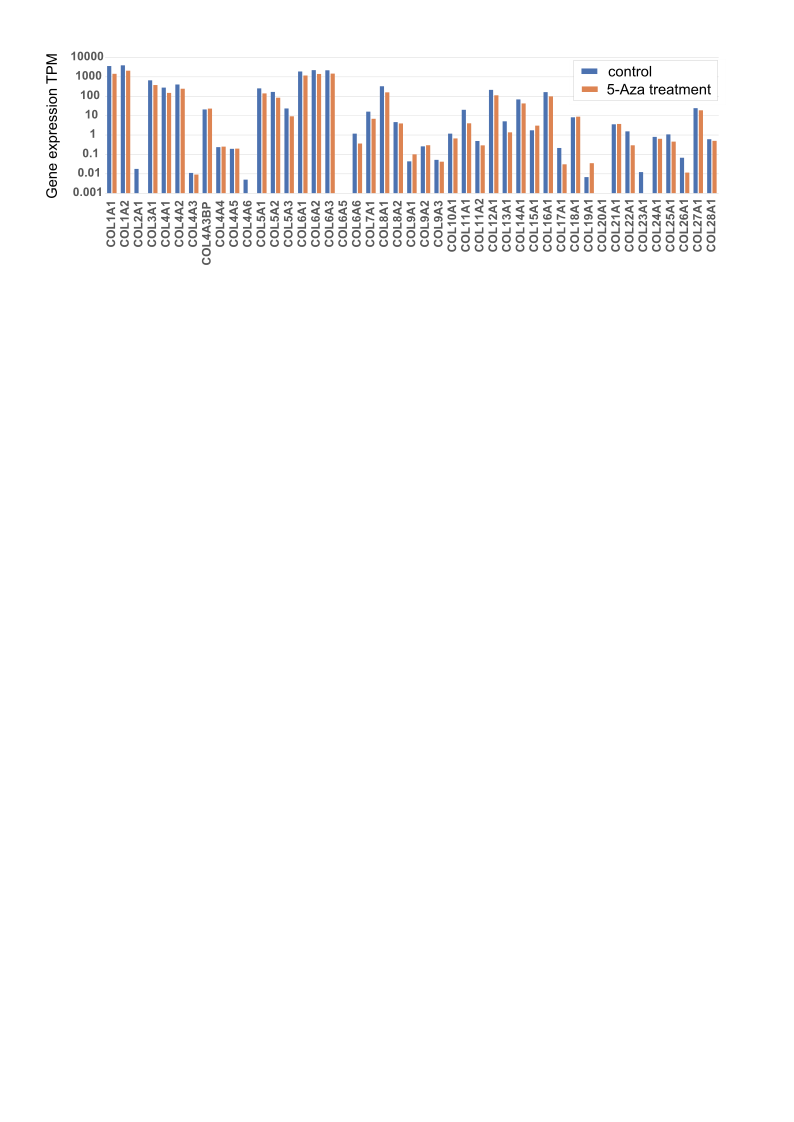

Supplement: Supplementary file 1 [file cells-15-00524-s001.zip › cells-4130628-supplementary-revised/Supplementary_Figure_S2_revised.png]
